# Detection of colorectal dysplasia using fluorescently labelled lectins

**DOI:** 10.1038/srep24231

**Published:** 2016-04-13

**Authors:** Joe Chin-Hun Kuo, Ashraf E. K. Ibrahim, Sarah Dawson, Deepak Parashar, William J. Howat, Kiran Guttula, Richard Miller, Nicola S. Fearnhead, Douglas J. Winton, André A. Neves, Kevin M. Brindle

**Affiliations:** 1 Cancer Research UK Cambridge Institute, University of Cambridge, Li Ka Shing Centre, Cambridge, UK; 2 Department of Biochemistry, University of Cambridge, Cambridge, UK; 3 Department of Pathology, Division of Molecular Histopathology, University of Cambridge, Addenbrooke’s Hospital, Cambridge, UK; 4 MRC, Laboratory of Molecular Biology, Hills Road, Cambridge, UK; 5 Cambridge Clinical Trials Unit, University of Cambridge, Cambridge, UK; 6 Statistics and Epidemiology Unit & Cancer Research Centre, Division of Health Sciences, Warwick Medical School, The University of Warwick, Coventry, UK; 7 Cambridge Colorectal Unit, Addenbrooke’s Hospital, Cambridge, UK

## Abstract

Colorectal cancer screening using conventional colonoscopy lacks molecular information and can miss dysplastic lesions. We tested here the ability of fluorescently labelled lectins to distinguish dysplasia from normal tissue when sprayed on to the luminal surface epithelium of freshly resected colon tissue from the *Apc*^*min*^ mouse and when applied to fixed human colorectal tissue sections. Wheat germ agglutinin (WGA) showed significantly decreased binding to adenomas in the mouse tissue and in sections of human colon from 47 patients. Changes in WGA binding to the human surface epithelium allowed regions containing normal epithelium (NE) or hyperplastic polyps (HP) to be distinguished from regions containing low-grade dysplasia (LGD), high-grade dysplasia (HGD) or carcinoma (C), with 81% sensitivity, 87% specificity and 93% positive predictive value (PPV). *Helix pomatia* agglutinin (HGA) distinguished epithelial regions containing NE from regions containing HP, LGD, HGD or C, with 89% sensitivity, 87% specificity and 97% PPV. The decreased binding of WGA and HPA to the luminal surface epithelium in human dysplasia suggests that these lectins may enable more sensitive detection of disease in the clinic using fluorescence colonoscopy.

Progression of colorectal cancer (CRC) from low- to high-grade dysplasia^1^ provides an opportunity for prophylactic removal^2^,^3^ of low-risk adenomas, which has been shown to reduce mortality^4^. However, colonoscopy misses >20% of adenomatous polyps^5^,^6^,^7^, including high-grade lesions in the proximal colon^8^,^9^. Moreover, in inflammatory bowel diseases (IBD) dysplasia may appear normal, requiring the entire colon to be randomly biopsied for effective surveillance^10^,^11^. Furthermore, colonoscopy cannot distinguish between dysplasia and hyperplasia, which is non-neoplastic and does not always require excision^12^, but frequently requires differentiation from dysplasia using biopsy or polypectomy. Since polypectomy carries a low risk of fatal bleeding and colon perforation^13^ there is a need for the development of methods that can distinguish hyperplasia from dysplasia at colonoscopy.

Targeted molecular imaging agents can enhance contrast between non-neoplastic and neoplastic tissues, improving the detection of dysplasia^14^. Fluorescently-labelled antibodies, injected intracardially in an animal model, and small peptides applied topically in patients, have enhanced detection of colon neoplasia using confocal laser microendoscopy (CLM)^15^,^16^. However, CLM has a microscopic field-of-view and can only examine small regions of the colon. Wide-field fluorescence imaging, which can be integrated into conventional endoscopes, could allow rapid screening of the entire colon. This has been achieved using small peptides labelled with near-infrared fluorophores and applied topically for the detection of colon neoplasia in animal models^17^. However, the molecular targets of these peptides are unknown, and therefore they may lack specificity.

Changes in glycosylation provide potential biomarkers of colon dysplasia^18^,^19^. Mucins cover the entire colonic mucosa and changes in their expression and glycosylation are associated with progression to CRC^20^,^21^ and can be associated with a poor prognosis^22^,^23^,^24^,^25^. Sialic acid content changes in colonic neoplasia^26^ and hyperplastic tissue secretes mucus rich in sialomucins^27^. Therefore imaging agents that bind specific glycan moieties may be useful in distinguishing normal from dysplastic tissues as well as hyperplasia from dysplasia.

We have shown previously that topically applied fluorescently labelled lectins can detect glycosylation changes in freshly resected oesophagus, potentially allowing endoscopic identification of oesophageal dysplasia^28^. Lectins are a family of glycan-specific proteins^29^ that are relatively non-toxic and inexpensive to produce. We have investigated here the potential of fluorescently labelled lectins to detect dysplasia elsewhere in the gastrointestinal tract, in this case the colon, using lectins that have been reported previously to show changes in binding to colorectal neoplasia, including *Helix pomatia* agglutinin (HPA)^30^, *Artocarpus integrifolia* or jackfruit lectin (JFL)^31^, *Arachis hypogaea* or peanut agglutinin (PNA)^32^, *Glycine max* or soybean agglutinin (SBA)^33^, and *Triticum vulgaris* or wheat germ agglutinin (WGA)^31^. Using freshly resected colon tissue from the *Apc*^*min*^ mouse and formalin fixed paraffin embedded (FFPE) human tissue sections, we show that some of these lectins, when fluorescently labelled, can distinguish between normal and dysplastic tissue from their differential binding to colorectal luminal surface epithelium. Since this surface is accessible to endoscopic examination, these lectins have the potential to be translated to the clinic for detecting colorectal dysplasia using fluorescence colonoscopy.

## Results

### Lectin binding to freshly resected intestines from the *Apc* ^*min*^ mouse

**Figure 1:**
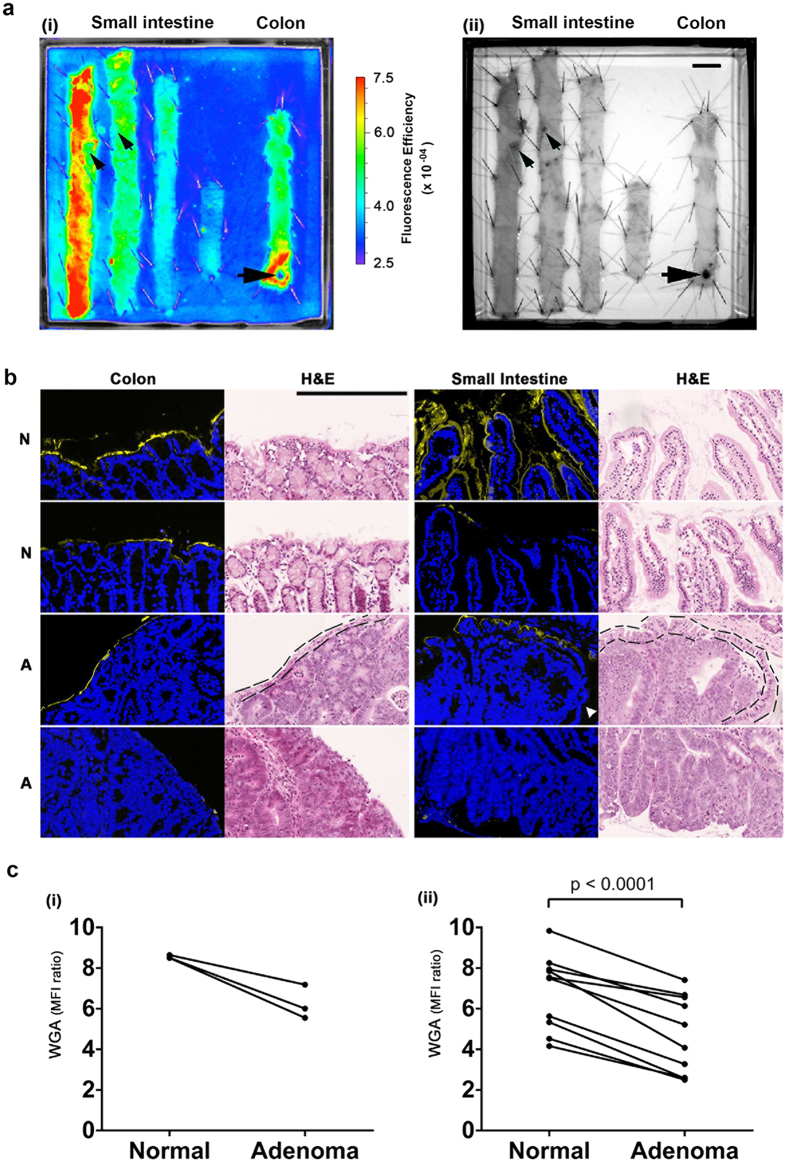
Images of freshly resected intestines from Apc^min^ mice that have been incubated with fluorescently-labelled WGA. **(a)** Macroscopic images; (i) far-red fluorescence image (Ex^**λ**^ = 615–665 nm, Em^**λ**^ = 695–770 nm); (ii) bright-field greyscale image. Black arrows indicate visible adenomas in the colon and small intestine ((i) and (ii)); intestines displayed in the proximal to distal direction, left to right on the plate. Scale bar in (ii) represents 1 cm. **(b)** Fluorescence microscopy of WGA binding to the luminal surface epithelium of normal intestine (N) and adenomas (A). WGA is shown in yellow in columns 1 and 3. Nuclei were stained with DAPI (in blue). The same sections were counter stained with haematoxylin and eosin (H&E) (columns 2 and 4). The dashed lines on the H&E stained images represent the normal cell layer that covered some adenomas in the colon and small intestine^35^. The white arrow indicates lack of WGA binding to a normal cell layer on the surface of an adenoma. Row 4 shows adenoma tissue with no overlying normal cell layer that is devoid of WGA fluorescence. Scale bar, 250 μm (row 1, column 2). **(c)** Regions of interest representing normal tissue and adenomas were analysed for WGA binding, which is expressed as the ratio of lectin mean fluorescence intensity versus the background fluorescence (MFI ratio), and averaged to give a score for each mouse (individual lines); (i) colon and (ii) small intestine. The P value is from a two-tailed paired t-test.

Fluorescently-labelled WGA was sprayed onto the luminal surface epithelium. Adenomas, which occur less frequently in the colon as compared to the small intestine in this animal model^34^, were easily identifiable (Fig. 1a(ii), black arrows). Macroscopically, WGA showed binding to normal colon epithelium (Fig. 1b; row 3, column 1) and decreased binding to adenomas (Fig. 1a(i), black arrows). Although lectin binding decreased with distance along the small intestine (Fig. 1a(i)) contrast between the adenomas and surrounding normal tissue was maintained. Microscopic examination confirmed that WGA fluorescence was limited to the luminal surface epithelium, as would be expected from topical application (Fig. 1b; columns 1 and 3). Unlike in the human disease, adenomas in the intestines from *Apc*^*min*^ mice can display a covering layer of normal epithelial cells (Fig. 1b; row 3)^35^. However, similar to what we observed subsequently in human colorectal tissue sections, WGA binding was diminished in adenomas in relation to normal tissue (Fig. 1b; row 4). Binding of WGA was quantified and expressed as the mean fluorescence intensity (MFI) ratio of lectin-to-background (Fig. 1c). This paired analysis showed a reduction in WGA binding to adenomas in the colon (Fig. 1c(i)), and similarly in the small intestine (Fig. 1c(ii)).

### Lectin binding to fixed tissue sections from human colon

**Figure 2:**
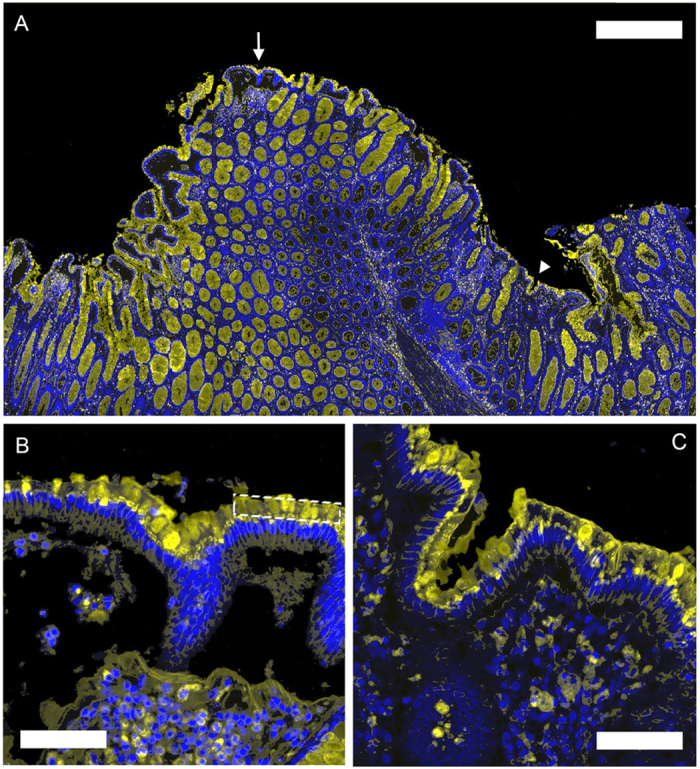
Binding of fluorescently labelled lectins to colorectal luminal surface epithelium. The figure shows a representative example of the binding of WGA conjugated to AF647 to colorectal tissue sections. **(a)** Luminal surface epithelium was defined as the sole region of interest (ROI), which would be visible at colonoscopy. **(b)** ROIs of defined length (ca. 500 μm) and thickness (20 μm) were defined at the luminal surface epithelium, using an automated image analysis system (Ariol™), as illustrated by the white dashed-line box. The insets **(b,c)** are 3.5 × magnifications of the ROIs indicated by the white arrow and triangle in **(a)**, respectively. Scale bars = 250 μm **(a)** and 70 μm **(b,c)** μm.

**Figure 3:**
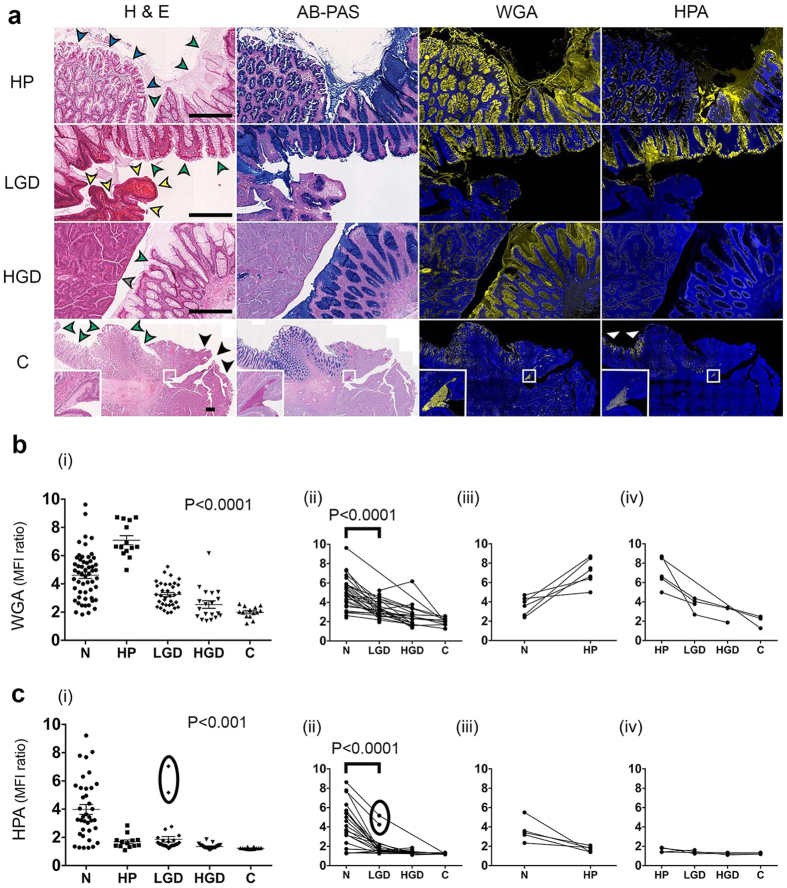
Quantitative analysis of fluorescently labelled lectin binding to colorectal luminal surface epithelium. **(a)** Colorectal tissue sections were stained with wheat germ agglutinin (WGA) or *Helix pomatia* agglutinin (HPA). Lectin binding to the different pathology classes is shown in yellow in columns 3 and 4 from left. The same sections were stained with a nuclear stain (DAPI, in blue in columns 3 and 4) and with haematoxylin and eosin (H&E; column 1). Regions of interest (in column 1) containing normal (N, green arrows), hyperplasia (HP, dark blue arrows; row 1), low-grade dysplasia (LGD, yellow arrows; row 2), high-grade dysplasia (HGD, grey arrows; row 3) and carcinoma (C, black arrows; row 4) are indicated. Alcian blue (AB) – periodic acid Schiff (PAS) combination stain (column 2) was applied to immediately adjacent tissue sections to visualize the presence of acidic (blue) and neutral (magenta) mucins. White arrows (column 4) indicate normal epithelium distant (>5 mm) from carcinoma. Insets in the carcinoma tissue sections (row 4) indicate WGA and HPA binding to PAS positive luminal necrosis as well as luminal malignant glands invading the bowel wall, deep within the carcinoma tissue. Scale bars (column 1), 1 mm. WGA **(b)** and HPA **(c)** binding to colorectal tissues was quantified as the ratio of mean lectin fluorescence intensity versus the background fluorescence (MFI ratio), which generated a score (*y*-axis) for the different pathology classes (*x*-axis), for each sample in patient-unmatched analyses **(b(i)**,**c(i))**. The *P* value represents the Jonckheere-Terpstra test for trend. These data were averaged to give a single score for each pathological class in each patient for the patient-matched analyses (ii-iv) for both WGA **(b)** and HPA **(c)**. Circled data points in **(c(i))** correspond to those in **(c(ii))**. Statistical significance (in **b(ii)**,**c(ii)**) was determined by Wilcoxon matched-pairs signed rank test.

Next, binding of fluorescently-labelled WGA and other lectins to the luminal surface epithelium of fixed human colon tissue sections was investigated (Fig. 2). Lectin binding was compared with histological assessment of paraffin-embedded colorectal samples derived from adenoma lesions collected from 47 patients. ROIs representative of the pathology classes present (Fig. 3a), were analysed (Fig. 3b(i) and Supplementary Fig. 1). Lectin fluorescence signals were averaged to give a score for each class. WGA and HPA binding showed significant differences across the different pathology classes (*P *< 0.001). WGA showed highest binding to hyperplasia (Fig. 3b(i)) and decreased binding in the progression from normal epithelium to dysplasia (LGD and HGD) and carcinoma (C). A similar trend was observed for HPA binding (Fig. 3c(i)), although HPA bound minimally to hyperplasia. Both lectins showed variable binding to normal epithelium (Supplementary Fig. 2c), which may reflect partial loss of mucus due to FFPE tissue processing. Normal epithelium, immediately adjacent to HGD or C, showed minimal HPA binding but this increased dramatically with distance (>5 mm) (white arrows, row 4, column 4 in Fig. 3a; Supplementary Fig. 2a). WGA binding showed no such differences (row 4, column 3 in Fig. 3a, Supplementary Fig. 2b). Soybean agglutinin (SBA) binding showed a significant decrease with disease progression (*P* = 0.05), however binding to all classes was relatively weak (Supplementary Fig. 1). Jackfruit lectin (JFL) and peanut agglutinin (PNA) also showed relatively low binding (Supplementary Fig. 1) and no significant trends were observed (*P* = 0.064 and *P* = 0.259 for JFL and PNA, respectively).

Lectin binding to samples from individual patients was averaged to give each pathology class a score for each patient (Fig. 3b(ii–iv) and, Supplementary Fig. 1). The same trend of decreased binding with disease progression was observed for WGA and HPA. Outliers that showed high HPA binding (circled in Fig. 3c(i,ii) were no longer outliers as these patients also showed very high mean HPA binding to normal epithelium (Fig. 3c(ii)).

### Analysis of lectin sensitivity and specificity

WGA and HPA showed good sensitivity and specificity in distinguishing non-dysplastic (normal or hyperplasia) from dysplastic epithelium (LGD or HGD, third row in Table 1). SBA, PNA and JFL showed low sensitivity and specificity (Table 1). WGA showed a remarkable ability to distinguish between hyperplasia and dysplasia or carcinoma (HP v LGD, HGD, C), with 100% sensitivity and 100% specificity, which was consistent with its increased binding to hyperplasia (Fig. 3b). These data suggest that fluorescently labelled WGA and HPA could potentially be used to distinguish normal from neoplastic luminal surface epithelium using fluorescence endoscopy, especially for severe lesions, and that WGA could be used to distinguish between hyperplastic and dysplastic polyps, in particular those with severe high-grade dysplasia.

### Mucin histochemistry

**Figure 4:**
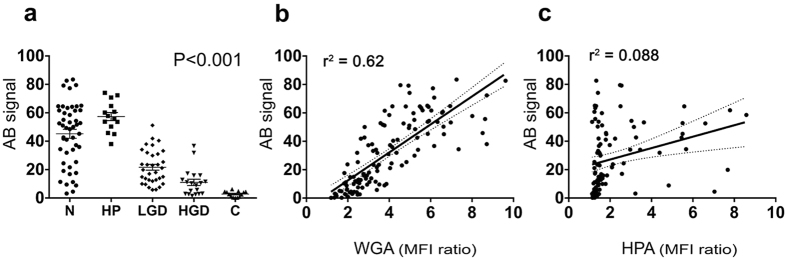
Quantitative analysis of acidic mucins on the luminal surface epithelium of the different colorectal pathology classes and correlation with lectin binding. (**a**) Tissue sections were stained with Alcian blue (AB)–periodic acid Schiff (PAS) combination stain and the resulting colour analysed for the presence of acidic mucins, by using a trained algorithm selective for the “blue” colour produced by AB staining (Fig. 3a, column 2). AB signals were averaged to generate scores (*y*-axis) for the different pathology classes (*x*-axis) in each sample in unmatched patient analyses. The *P* value represents the Jonckheere-Terpstra test for trend. Abbreviations: N, normal; HP, hyperplasia; LGD, low-grade dysplasia; HGD, high-grade dysplasia; C, carcinoma. Linear regression analysis of acidic mucin staining, as a function of WGA (**b**) and HPA (**c**) fluorescence, for unmatched patient analyses. WGA, wheat germ agglutinin. HPA, *Helix pomatia* agglutinin. Dashed lines (**b**,**c**) represent the 95% confidence interval hyperbolas for the linear best fits (solid lines).

Alcian blue (AB)–periodic-acid Schiff (PAS) combination stain (AB-PAS) was used to determine whether WGA and HPA were binding to acidic mucins (sialomucins and sulfomucins) stained blue by AB, or neutral mucins, stained deep-red/magenta by PAS (Fig. 3). The AB staining pattern closely resembled that observed for WGA, but not HPA (Figs 3 and 4). Regions of hyperplasia showed the strongest staining of luminal surface epithelium, with decreased staining in the progression from normal tissue to dysplasia and carcinoma (Fig. 4a) (P* *< 0.001). There was a strong correlation between WGA binding and AB staining (*R* = 0.79, *P *< 0.0001, Pearson product moment correlation; Fig. 4b), consistent with the specificity of WGA for acidic glycans (sialic acids)^33^. Significant correlations between WGA binding and AB staining were observed for normal epithelium, LGD and HGD but not for HP and carcinoma (Supplementary Table 2a). AB staining, similarly to WGA binding, could distinguish between non-neoplastic epithelium (normal or hyperplasia) and neoplasia with high sensitivity and specificity (Supplementary Table 3). As with WGA binding, normal epithelium showed a wide range of AB staining (Fig. 4a), which again might be explained by loss of mucus during tissue processing. There was no correlation between HPA binding and AB staining, consistent with HPA’s lack of specificity for acidic glycans^34^ (Fig. 4c and Supplementary Table 2). PAS staining was weak across all the classes and showed no correlation with disease progression (*P* = 0.525, Supplementary Fig. 3a) or with WGA or HPA binding (Supplementary Fig. 4b,c, Supplementary Table 2b). Deposits of PAS positive material were observed within the lumen of HGD and C (row 4, column 2 in Fig. 3a, Supplementary Fig. 4) and appeared to correlate with glandular regions that showed very strong WGA binding (row 4, column3 in Fig. 3a). These regions occurred mostly deep beneath the luminal surface epithelium and therefore were excluded from analysis. The luminal surface epithelium of HGD and C were mostly devoid AB-PAS staining (column 2, row 3 and 4 in Figs 3a and 4a, Supplementary Fig. 3a).

## Discussion

Fluorescently labelled WGA sprayed onto the luminal surface of freshly resected intestines from the *Apc*^*min*^ mouse showed decreased binding to adenomas. This is in agreement with the reported reduced staining by WGA of glycosylated mucus proteins, particularly mucin 2 (Muc2), in the *Apc*^*min*^ mouse^36^. Muc2 expression is known to be down regulated in both mouse^37^ and human^38^ colorectal tumours when compared to healthy colonic tissue. This suggested to us that spraying of WGA onto the luminal surface of the human colon *in situ*, when used in conjunction with fluorescence colonoscopy, would have the potential to enhance detection of dysplasia, as we have demonstrated previously for the oesophagus^28^. To investigate relevance to the human disease we analysed the capability of fluorescently labelled WGA and other lectins to distinguish dysplastic or neoplastic surface epithelium from normal or hyperplastic surface epithelium in fixed sections of human colon. WGA distinguished epithelial regions containing NE or HP from regions containing LGD, HGD or carcinoma, with 81% sensitivity, 87% specificity and 93% positive predictive value (PPV). HPA distinguished epithelial regions containing NE from regions containing HP, LGD, HGD or carcinoma, with 89% sensitivity, 87% specificity and 97% PPV.

Lectin binding to abnormal and diseased colorectal epithelium has been studied extensively in the past using conventional lectin histochemistry methods. However, these studies typically focused on binding to cross sections of the colonic mucosa as a whole rather than specifically to the luminal surface epithelium^32^,^39^,^40^. We have shown here that the luminal surface epithelium of high-grade dysplasia (HGD) and carcinoma is largely devoid of mucus and low in lectin binding. Conversely, deep beneath the mucosal surface, the glandular lumen of these lesions appear to contain material that stains with periodic-acid Schiff (PAS), which detects neutral mucins, and which binds all the lectins studied here (Fig. 3a and Supplementary Fig. 4). This may represent mucus secreted by these advanced lesions.

Limitations of the study include the lower numbers of adenomas in the colon when compared with the small intestine in the *Apc*^*min*^ mouse model and some loss of luminal surface mucus in the human FFPE material. The latter was evident when staining normal tissues with AB (Supplementary Fig. 2c). This surface mucus, which was responsible for the observed binding of WGA to normal epithelium, is lost in advanced dysplasia and carcinoma^41^,^42^. The luminal surface mucus may be better preserved using alcohol fixation methods or by using frozen tissue sections^41^ or ideally freshly resected unfixed colon samples, as were used for the studies with intestines from *Apc*^*min*^ mice (Fig. 1). Fresh colorectal tissue sections have a thicker mucus layer than their fixed counterparts^41^,^43^.

A further limitation was the limited sample size of the patient-paired data. Samples from each individual patient often did not contain more than two pathological classes, reflecting the limited heterogeneity of sporadic CRC. Therefore, even though the patient-matched analyses confirmed the trends observed for unmatched data (Fig. 3b,c), the analysis of significance, sensitivity and specificity could not be determined in patient-matched data.

The presence of acidic mucins, stained by AB, showed a significant decrease with disease progression, similar to that shown by WGA binding (Fig. 4), which can be explained by the specificity of WGA for sialic acid^44^, a major terminal moiety of acidic mucins. There was no significant trend for PAS staining, indicating no differences in neutral mucin content on the luminal surface epithelium, and there was no correlation with lectin binding (Supplementary Fig. 3).

The removal of LGD at colonoscopy is crucial for reducing mortality in sporadic CRC^4^. Moreover, in patients with IBD, LGD can occur as flat mucosal lesions, which are often difficult to detect at colonoscopy^45^. Although in the cohort analysis WGA and HPA showed low sensitivity for distinguishing normal and LGD (0.57 for WGA and 0.33 for HPA; Supplementary Table 1), in the patient-matched data, decreased binding was observed between normal and LGD in all patients for WGA (n = 16; *P *< 0.0001 by Wilcoxon test) and in all but one for HPA (*P *< 0.0001 by Wilcoxon test). WGA binding could also distinguish between hyperplasia and neoplasia, which is explained by its binding to sialic acids^44^.

HPA has specificity for alpha-*N*-acetylgalactosamine (α-GalNAc), the immune determinant sugar of histo-blood group A^46^. For this reason, HPA may react differently according to ABO blood group type. We have found this not to be the case here, as normal colon sections from patients with different blood group types (Fig. 3; Supplementary Table 4) did not show differential staining with HPA (Supplementary Fig. 5).

Hyperplasia is often accompanied by an increase in acidic mucins that have high levels of sialic acids (sialomucins)^27^, a trend also observed in this study (Fig. 4). Hyperplastic and dysplastic polyps are difficult to distinguish in routine endoscopy, due to their similar appearance, and as a consequence all suspicious polyps of a minimal size (>5 mm) are resected. Moreover, hyperplastic polyps (HP), which have reduced potential for malignant transformation^12^, can appear similar at colonoscopy to sessile serrated adenomas (SSA), which can be precursors to CRC^47^,^48^. Nevertheless, the removal of small polyps during endoscopy still carries a risk of colon bleeding and perforation^13^. Fluorescently labelled WGA and HPA have the potential to identify dysplastic polyps and to distinguish them from hyperplasia or normal tissue.

We have shown previously that fluorescently labelled lectins can be used for endoscopic identification of dysplasia in Barrett’s oesophagus^28^. Similarly to what was observed here, WGA and HPA showed high binding to oesophageal mucosa and Barrett’s and low binding to dysplastic tissue. Although the contrast observed here in the colon and previously in the oesophagus is negative, this is not an issue in the context of endoscopic surveillance since any regions with confounding factors that lead to loss of binding (false positives) would inevitably be biopsied. In contrast, confounding factors that lead to loss of binding of an imaging agent that generates positive contrast, i.e. that binds to diseased areas (false negatives), could result in failure to detect the presence of disease.

Lectins of plant or animal origin are potentially toxic. However, both WGA and HPA are components of foodstuffs, wheat germ and edible snail, respectively, and in the case of WGA, part of a basic, gluten-containing diet. Moreover, most studies that have investigated lectin toxicity have been conducted using much higher lectin doses than those used here and over much longer periods of time^49^. Therefore we do not anticipate any toxicity with the use of these lectins. Furthermore, any potential toxicity could be reduced by washing off the lectin with a large molar excess of a lectin-binding monosaccharide (GlcNAc for WGA and GalNAc for HPA) following the imaging session^28^.

In conclusion, fluorescently-labelled lectins, particularly, WGA or HPA, may be useful in the secondary surveillance setting of sporadic CRC, to enhance detection of dysplasia using fluorescence colonoscopy and, in particular with WGA, to allow hyperplasia to be distinguished from dysplasia.

## Methods

### Apc^min^ mice

All experiments were conducted in accordance with the Animals (Scientific Procedures) Act of 1986 (United Kingdom) and were designed with reference to the UK Co-ordinating Committee on Cancer Research Guidelines for the Welfare of Animals in Experimental Neoplasia. The work was approved by the Cancer Research UK Cambridge Institute Ethical Review Committee. The small intestine, caecum and colon were removed post mortem from *Apc*^*min*^ mice (n = 10) aged between 120–140 days, flushed with ice-cold blocking buffer (PBS containing 1% foetal bovine serum, FBS), incubated for 15 min with AlexaFluor™−647 (AF647) conjugated WGA (Life Technologies, Paisley, UK) at 5 μg/ml, by clamping the two ends of the intestines at 20 °C. The clamped intestines were immersed in PBS during incubation to avoid dehydration and subsequently flushed once with ice-cold blocking buffer. A rapid fixation was then performed, by flushing the intestines with 10% neutral buffered formalin (NFB; 4% Formaldehyde in PBS, Sigma-Aldrich, Buchs SG, Switzerland). After a further wash with ice-cold PBS, the intestines were sectioned, dissected and pinned luminal side uppermost on a wax plate and imaged using an IVIS200™ camera (Perkin Elmer, Hopkinton, MA, USA), with a Cy55 filter set (Ex^**λ**^ = 615–665 nm, Em^**λ**^ = 695–770 nm). The intestines were then fixed for 24 h with 10% NFB, replaced with 70% ethanol for 24 h at 4 °C and subsequently processed and embedded in paraffin blocks. Tissue sections were mounted using ProLong™ Gold Anti-fade reagent with DAPI (Life Technologies) for 24 h at room temperature, and examined by fluorescence microscopy, using a 20× lens, producing a mosaic of images that captured the entire tissue section. Fluorescence micrographs were analysed using an Ariol™ imaging system (Leica Microsystems Ltd, Milton Keynes, UK). AF647 fluorescence was false-coloured in yellow.

### Human samples

Colonoscopy biopsies or colonic resections were performed between 2008 and 2012 (Supplementary Table 4). Lectin binding was compared with histological assessment on 100 formalin-fixed paraffin-embedded (FFPE) colorectal samples derived from adenoma lesions collected from 47 patients (32 males, 15 females; mean age of 68.8 ± 8.6 yr., range 53–95 yr.). Informed written consent was obtained from all subjects. Approval was obtained from a local ethics committee (Cambridgeshire Local Research Ethics Committee, CLREC, ref. 06/Q0108/307). All the histological procedures were carried out in accordance with the guidelines approved by the CLREC. All experimental protocols were approved by the CLREC. Normal epithelium (NE) occupied 38.1% of the area of the tissue sections, hyperplastic polyps (HP) 16.1%, low-grade (LGD) dysplasia 24.4%, high-grade (HGD) dysplasia 13.1%, and carcinoma (C) 8.3%. Mean lesion size was 16.0 ± 14.2 mm (range 2–60 mm), located in the caecum (3%), ascending (17%), transverse (14%), descending (11%) and sigmoid (35%) colon and in the rectum (20%). H&E-stained, colorectal tissue sections (5-μm), from 47 patients, were reviewed by a senior histopathologist (A.I.), and identified as normal colon (NE; n = 64), hyperplasia (HP; n =27), low-grade dysplasia (LGD; n = 41), high-grade dysplasia (HGD; n = 22) or carcinoma (C; n = 14).

### Lectin histochemistry

AF647 conjugated lectins (Life Technologies) were used on a Duolink™ (Olink Bioscience, Uppsala, Sweden) system with Shandon Sequenza™ racks and cover plates (Thermo Fisher Scientific, Waltham, MA, USA). Deparaffinised slides, washed and blocked at 4 °C using lectin binding buffer (LBB; 20 mM HEPES, 150 mM NaCl, 1 mM CaCl_2_, MgCl_2_ and MnCl_2_, and 1% FBS, pH 7.4), were stained with lectin (5 μg/ml) for 15 min at 37 °C and then washed in cold LBB buffer, then LBB buffer with no serum, before mounting with ProLong™ Gold with DAPI. Fluorescence was imaged using a 20× lens that captured the entire tissue section. For ease of visualization, AF647 and DAPI fluorescence were false-coloured in yellow and blue, respectively.

### Alcian blue - periodic acid Schiff combination staining

Deparaffinised slides were incubated in Alcian Blue (AB) (pH 2.5) for 10 min, washed in water, incubated in 0.5% periodic acid Schiff reagent (PAS) for 5 min, washed in water, and further incubated in PAS (Thermo Fisher Scientific) for 15 min and then washed in water. The slides were counterstained in Mayers Haematoxylin for 45 s, rinsed with water, dehydrated through 2 changes of 100% ethanol and cleared with 2 changes of xylene and mounted with DPX mountant (Sigma-Aldrich). Slides were scanned into an Ariol™ imaging system and regions of interest (ROI) within 20 μm of the luminal surface epithelium were defined (Fig. 2). Lectin binding to slide surface that was not covered by tissue was defined as background. AB – PAS staining was analysed using a trained algorithm optimised for quantifying the AB and PAS signals. Average ROI intensities were normalised against background signal.

### Statistics

The significance of lectin binding and AB and PAS staining were assessed using the Jonckheere-Terpstra test, using 5000 permutations to calculate the reference distribution. As some patients had multiple samples but few had complete data, bootstrapping was used to repeatedly sample the data to ensure that all the data were used whilst maintaining the assumption of independence. 1000 bootstraps were used for lectin, AB and PAS staining and the median P-value over the 1000 bootstraps was taken. Recursive partitioning was used to provide cut-offs in lectin binding to the different stages of disease progression. Predicted disease stages were compared with the true disease stages in terms of sensitivity and specificity, as well as positive and negative predictive values.

## Additional Information

**How to cite this article**: Kuo, J. C.-H. *et al.* Detection of colorectal dysplasia using fluorescently labelled lectins. *Sci. Rep.* **6**, 24231; doi: 10.1038/srep24231 (2016).

## Supplementary Material

Supplementary Information

## Figures and Tables

**Table 1:** Statistical analysis of lectin performance in distinguishing dysplastic or neoplastic from non-neoplastic tissues.

*Comparison*	*Sensitivity*(*n*)					*Specificity*				
	*HPA*	*JFL*	*PNA*	*SBA*	*WGA*	*HPA*	*JFL*	*PNA*	*SBA*	*WGA*
*N v* (*HP, LGD, HGD, C*)	0.89 (88)	0.22 (99)	0.62 (96)	0.50 (91)	0.65 (97)	0.87	0.67	0.73	0.76	0.77
*N v* (*LGD, HGD, C*)	0.85 (65)	0.73 (64)	0.73 (62)	0.76 (74)	0.81 (81)	0.89	0.25	0.62	0.63	0.79
*HP v* (*LGD, HGD + C*)	0.91 (61)	0.91 (51)	0.84 (61)	0.95 (51)	1.00 (54)	0.24	0.44	0.39	1.00	1.00
(*N, HP*) *v* (*LGD, HGD*)	0.69 (63)	0.61 (74)	0.50 (60)	0.64 (72)	0.71 (72)	0.59	0.61	0.63	0.82	0.87
(*N, HP*) *v* (*LGD, HGD, C*)	0.78 (88)	0.68 (99)	0.64 (96)	0.74 (91)	0.81 (93)	0.59	0.54	0.60	0.79	0.87
	*Positive predictive value*					*Negative predictive value*				
*N v* (*HP, LGD, HGD, C*)	0.97	0.74	0.92	0.81	0.87	0.64	0.17	0.27	0.43	0.48
*N v* (*LGD, HGD, C*)	0.95	0.68	0.88	0.86	0.93	0.70	0.29	0.38	0.48	0.56
*HP v* (*LGD, HGD, C*)	0.50	0.70	0.50	0.78	1.00	0.75	0.78	0.77	1.00	1.00
(*N, HP*) *v* (*LGD, HGD*)	0.55	0.49	0.44	0.81	0.88	0.73	0.72	0.69	0.66	0.69
(*N, HP*) *v* (*LGD, HGD, C*)	0.73	0.57	0.69	0.86	0.93	0.67	0.65	0.55	0.64	0.69

Lectin binding to non-neoplastic colon epithelium (N, normal and/or HP, hyperplasia) was compared with dysplasia (LGD, low-grade or HGD, high-grade dysplasia) or dysplasia grouped with neoplasia (C, carcinoma). Sensitivity is the probability of a positive test result given the patient really has the disease. Specificity is the probability of a negative test result in the true absence of disease. Positive predictive value is the probability that a patient with a positive test result has the disease. Negative predictive value is the probability that a patient with a negative test result does not have the disease. Abbreviations: HPA, *Helix pomatia* agglutinin; JFL, jackfruit lectin; PNA, peanut agglutinin; SBA, soybean agglutinin; WGA, wheat germ agglutinin. Values in parenthesis are sample numbers.
