# Supplementary material for: Detection of colorectal dysplasia using fluorescently labelled lectins
Source: Sci Rep. 2016 Apr 13;6:24231. doi: 10.1038/srep24231 (PMC4829854; doi:10.1038/srep24231)
Supplement: Supplementary Information [file srep24231-s1.pdf]

SUPPLEMENTARY INFORMATION

Detection of colorectal dysplasia using fluorescently labelled lectins

Joe Chin-Hun Kuo[1], Ashraf E K Ibrahim[3,4], Sarah Dawson[5], Deepak Parashar[6], William J Howat[1], Kiran Guttula[3], Richard Miller[7], Nicola S Fearnhead[7], Douglas J Winton[1], André A Neves*[1] & Kevin M Brindle[1,2]

[1]Cancer Research UK Cambridge Institute, University of Cambridge, Li Ka Shing Centre, Cambridge, UK; [2]Department of Biochemistry, University of Cambridge, Cambridge, UK; [3]Department of Pathology, Division of Molecular Histopathology, University of Cambridge, Addenbrooke's Hospital, Cambridge, UK; [4]MRC, Laboratory of Molecular Biology, Hills Road, Cambridge, UK; [5]Cambridge Clinical Trials Unit, University of Cambridge, Cambridge, UK; [6]Systems Biology and Oncology, Cancer Research Unit, Division of Health Sciences, Warwick Medical School, University of Warwick, Coventry, UK; [7]Cambridge Colorectal Unit, Addenbrooke's Hospital, Cambridge, UK.

Correspondence should be addressed to A.A.N (andre.neves@cruk.cam.ac.uk)

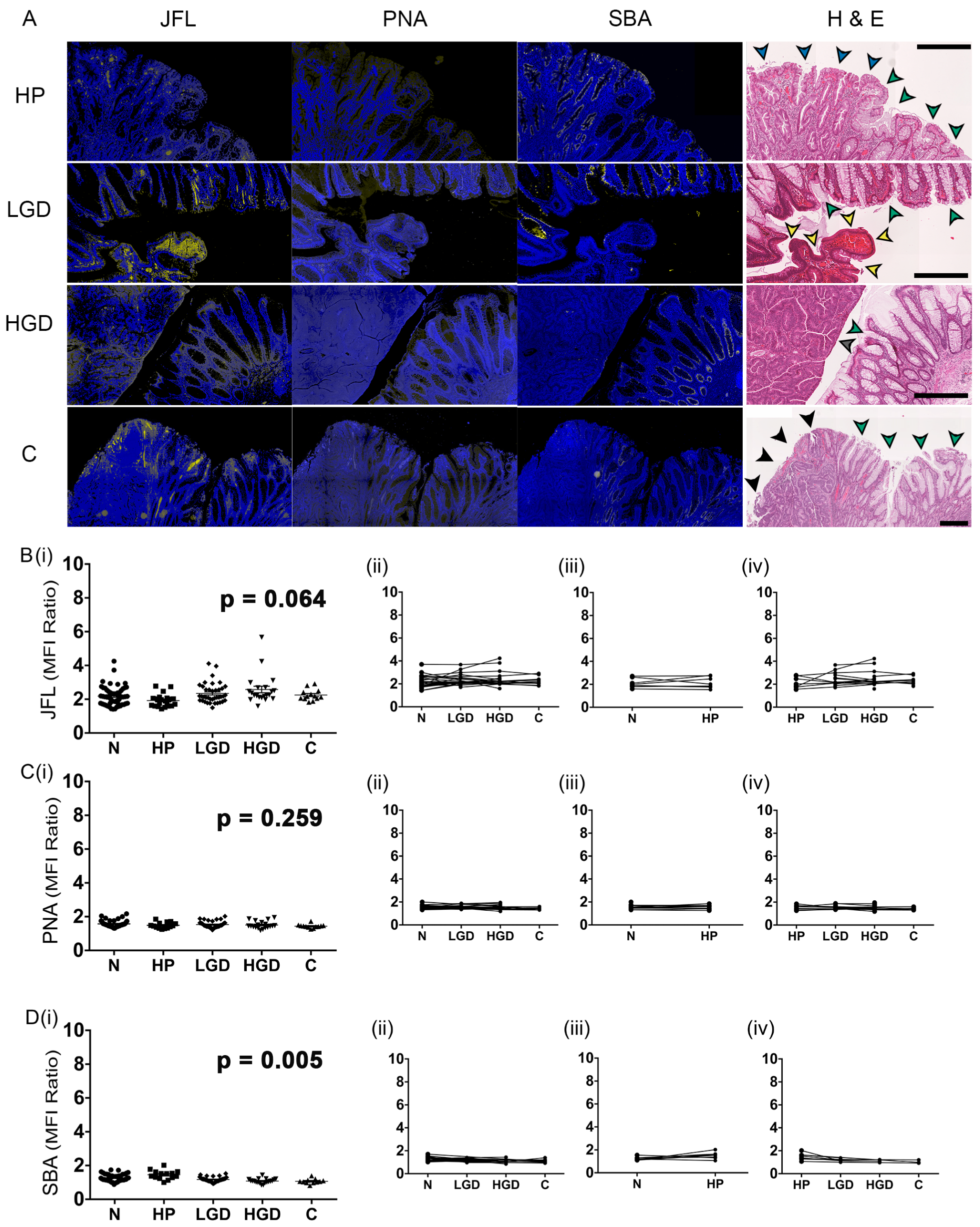

Supplementary Figure 1 Quantitative analysis of fluorescently-labelled lectin binding to colon luminal surface epithelium. (A) Paraffin-embedded tissue sections were stained with jackfruit lectin (JFL), peanut agglutinin (PNA) and soybean agglutinin (SBA) (AlexaFluor™ 647 conjugates, yellow; DAPI, blue). Hematoxylin and eosin staining (H&E) was used to identify the presence of disease: normal (N, green arrows), hyperplasia (HP, dark blue arrows), low- grade dysplasia (LGD, yellow arrows), high-grade dysplasia (HGD, grey arrows) and carcinoma (C, black arrows). Lectin fluorescence signals were averaged and normalized to the background for the different stages of disease. B (i), C (i) and D (i) show unmatched analyses for JFL, PNA and SBA respectively. Patient-matched analyses are shown in (ii), (iii) and (iv). The interconnected data points represent the averaged lectin fluorescence signals for the different disease classes in a single patient sample. Scale bars (H&E), 1 mm. The p value represents the Jonckheere-Terpstra test for trend.

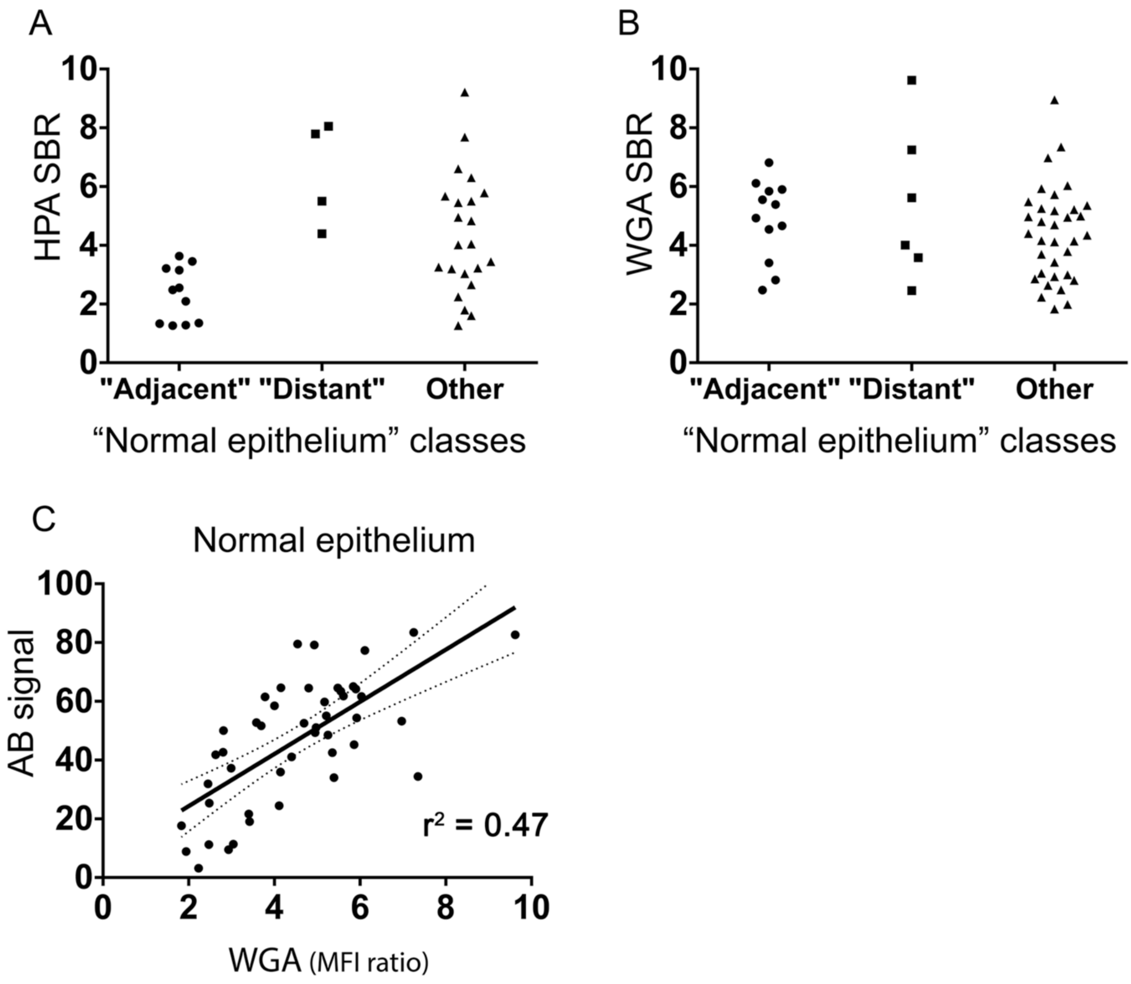

Supplementary Figure 2 Lectin binding to normal epithelium. (A) *Helix pomatia* agglutinin (HPA) and (B) wheat germ agglutinin (WGA) binding to normal epithelium “Adjacent” to or “Distant” (> 5 mm) from advanced disease (high grade dysplasia and carcinoma). “Other” represents unclassified normal epithelium where there were either no advanced lesions present or the proximity to such lesions was unknown. (C) Linear regression analysis of Alcian blue (AB) staining as a function of WGA fluorescence, for normal epithelium, demonstrating the wide variation in the mucus layer of normal epithelium between different samples.

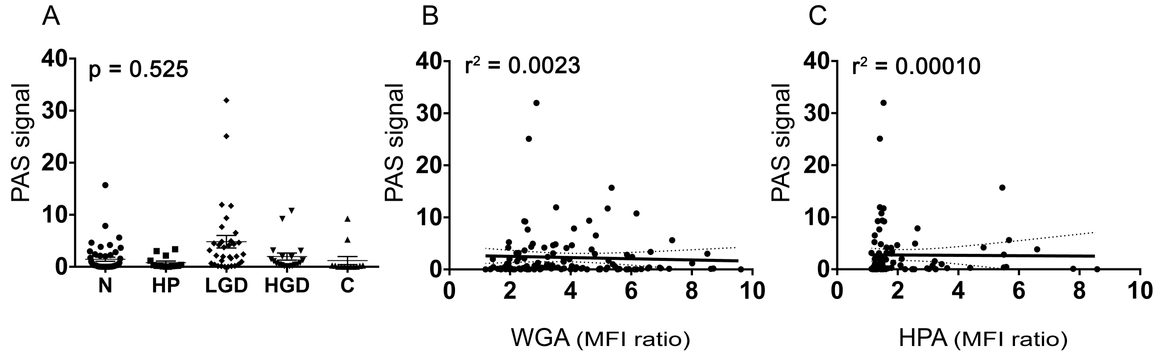

Supplementary Figure 3 Quantitative analysis of neutral mucin on the luminal surface colon epithelium for the different pathology classes, and correlation with lectin fluorescence. (A) Paraffin tissue sections were stained with Alcian Blue (AB) – periodic acid Schiff (PAS) combination stain and the PAS signal quantified using an algorithm classifier based on a mask selective for the 'magenta' color due to PAS staining (Figure 2A). PAS signals were averaged to give scores for the different pathology classes (x-axis), for each sample in unmatched patient analyses. The p value represents the Jonckheere-Terpstra test for trend. N, normal; HP, hyperplasia; LGD, low-grade dysplasia; HGD, high-grade dysplasia; C, carcinoma. Linear regression analysis of PAS signal as a function of WGA (B) and HPA (C) fluorescence. Dashed lines (B, C) represent the 95% confidence interval hyperbolas for the linear best fits (solid lines). Abbreviations: WGA, wheat germ agglutinin; HPA, *Helix pomatia* agglutinin.

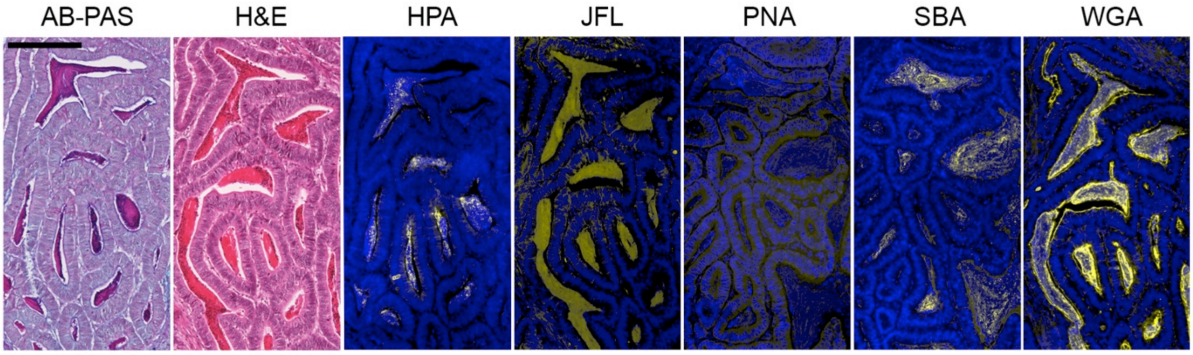

Supplementary Figure 4 Deep luminal mucous deposits within carcinoma tissue show lectin staining. Serial paraffin tissue sections were stained with Alcian Blue (AB) – periodic acid Schiff (PAS), hematoxylin and eosin (H&E) and with fluorescent lectins. Mucous deposits that are PAS positive show differential binding of several of the lectins. Abbreviations: *Helix pomatia* agglutinin; JFL, jackfruit lectin; PNA, peanut agglutinin; SBA, soybean agglutinin; WGA, wheat germ agglutinin. Scale bar (AB-PAS), 500 μm.

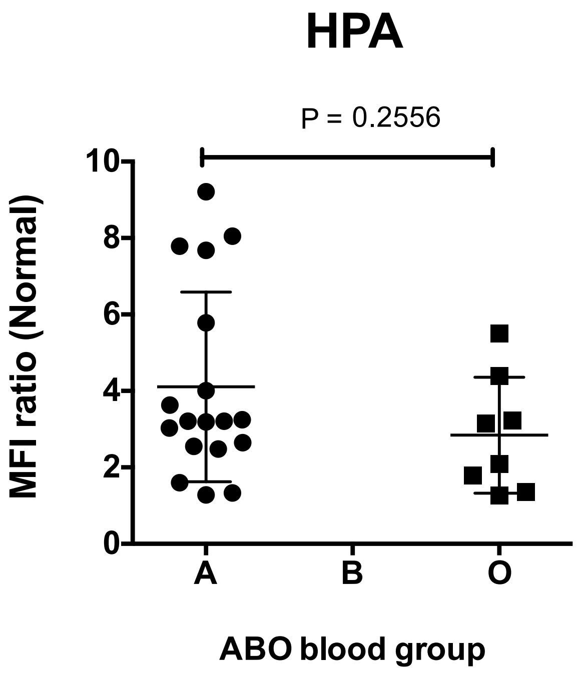

Supplementary Figure 5 Analysis of HPA lectin binding to normal colon, as a function of ABO blood group type. No difference was found between HPA staining to sections from patients with group A or group O. N=41 sections from 24 patients for which ABO blood group data was available. MFI – mean fluorescence intensity. p value, Mann-Whitney test.

Supplementary Table 1 Statistical analysis of lectin performance in distinguishing dysplastic or neoplastic from non-neoplastic tissues. Lectin binding to non-neoplastic colon epithelium (N, normal or HP, hyperplasia) was compared with binding to dysplasia (LGD, low grade or HGD, high grade dysplasia) or grouped with neoplasia (C, carcinoma). Sensitivity is the probability of a positive test result when the patient truly has disease. Specificity is the probability of a negative test result when the patient does not have disease. Positive predictive value is the probability that a patient with a positive test result truly has disease. Negative predictive value is the probability that a patient with a negative test does not have the disease. Abbreviations: HPA, *Helix pomatia* agglutinin; WGA, Wheat germ agglutinin.

Comparison	*Sensitivity (n)*		*Specificity*	
	HPA	*WGA*	*HPA*	*WGA*
N v LGD	0.33 (24)	0.57 (41)	0.89	0.83
N v HGD	0.88 (24)	0.60 (21)	1.00	0.94
N v (LGD, HGD)	0.78 (41)	0.71 (60)	0.89	0.79
LGD v (HGD, C)	0.76 (40)	0.77 (50)	0.67	0.68
	Positive predictive value		*Negative predictive value*	
N v LGD	0.50	0.81	0.80	0.60
N v HGD	1.00	0.75	0.94	0.88
N v (LGD, HGD)	0.90	0.88	0.76	0.56
LGD v (HGD, C)	0.97	0.80	0.18	0.65

Supplementary Table 2 Correlation between lectin fluorescence and the presence of (A) acidic mucins and (B) neutral mucins in the different colon pathology classes. Lectin fluorescence signal from labeled wheat germ agglutinin (WGA) or *Helix pomatia* agglutinin (HPA) was plotted against acidic mucin staining with Alcian blue (AB) in (A) and neutral mucin staining with periodic acid Schiff (PAS) in (B). Abbreviations: N, Normal; HP, hyperplasia; LGD, low-grade dysplasia; HGD, high-grade dysplasia; C, carcinoma.

A

Colon classes	*WGA v.s. AB*			*HPA v.s. AB*		
	Pearson r	*P value (two-tailed)*	*Number of XY Pairs*	*Pearson r*	*P value (two-tailed)*	*Number of XY Pairs*
N	0.69	< 0.0001	46	-0.07	0.71	30
HP	-0.37	0.20	14	0.49	0.10	12
LGD	0.56	0.0012	30	-0.34	0.08	27
HGD	0.80	< 0.0001	18	0.33	0.18	18
C	0.47	0.10	13	0.29	0.38	11
ALL	0.79	< 0.0001	120	0.23	0.02	98

B

Colon classes	*WGA v.s. PAS*			*HPA v.s. PAS*		
	Pearson R	*P value (two-tailed)*	*Number of XY Pairs*	*Pearson R*	*P value (two-tailed)*	*Number of XY Pairs*
N	0.01143	0.9399	46	0.2493	0.1922	29
HP	0.1539	0.5993	14	-0.2759	0.4116	11
LGD	0.05306	0.7807	30	0.1862	0.3727	25
HGD	0.6187	0.0062	18	0.15	0.5657	17
C	0.3066	0.3083	13	0.5352	0.0898	11
ALL	-0.04916	0.5939	120	0.03014	0.7742	93

Supplementary Table 3 Performance assessments of Alcian blue (AB) and periodic acid Schiff (PAS) staining for distinguishing between the different colon pathology classes. Sensitivity is the probability of a positive test result when the patient truly has disease. Specificity is the probability of a negative test result when the patient does not have disease. Positive predictive value is the probability that a patient with a positive test result truly has the disease. Negative predictive value is the probability that a patient with a negative test result does not have the disease. Abbreviations: PPV, positive predictive value; NPV, negative predictive value; N, normal; HP, hyperplasia; LGD, low-grade dysplasia; HGD, high-grade dysplasia; C, carcinoma;

Comparison	*Sensitivity (n)*		*Specificity*		*PPV*		*NPV*	
	AB	*PAS*	*AB*	*PAS*	*AB*	*PAS*	*AB*	*PAS*
N v LGD	0.80 (29)	0.78 (15)	0.86	0.67	0.86	0.78	0.80	0.67
N v HGD	0.83 (19)	0.36 (21)	0.92	0.40	0.83	0.40	0.92	0.36
N v (LGD+HGD)	0.87 (46)	0.70 (42)	0.80	0.33	0.90	0.72	0.75	0.31
N v (LGD,HGD+C)	0.89 (62)	0.64 (59)	0.80	0.33	0.93	0.79	0.71	0.19
HP v (LGD,HGD+C)	1.00 (45)	0.91 (44)	1.00	0.36	1.00	0.57	1.00	0.81
(N+HP) v (LGD+HGD)	0.87 (61)	0.66 (56)	0.90	0.50	0.90	0.64	0.87	0.52
LGD v (HGD+C)	0.77 (42)	0.50 (30)	0.75	0.70	0.83	0.77	0.66	0.41

Supplementary Table 4 Breakdown of patient cohort used in this study. The tissue samples used in the study were from patients who underwent colonoscopy/colonic resections between 2008 and 2012. These samples were obtained from the Histopathology Department and the Tissue Bank facility within Addenbrooke's Hospital (Cambridge, UK). All patients had given their consent for their tissue samples to be used for research including genetic research. Ethical approval for all the work conducted was obtained from the Cambridgeshire local research ethics committee (LREC ref. 06/Q0108/307).

Patients	Section	Description of section	Types on section					Age	Gender	Blood group	Site	Cancer type	pT	pN	pM	Dukes
			N	HP	LGD	HGD	C									
J1	J1-A	Polypectomy	x		x	x		76	f	A-	Sigmoid colon					
J2	J2-A	Biopsy of polyp				x		53	m	NK	Descending colon					
J3	J3-A	Polypectomy		x				68	m	NK	Sigmoid colon					
J3	J3-B	Biopsy of polyp		X				68	m	NK	Transverse colon					
J4	J4-A	Biopsy of polyp	x		x			72	m	NK	Descending colon					
J5	J5-A	Polypectomy	x			x		72	f	NK	Sigmoid colon					
J6	J6-A	Normal mucosal biopsy	x					95	m	O+	Descending colon					
J6	J6-B	Polypectomy		x				95	m	O+	Sigmoid colon					
J6	J6-C	Biopsy of cancer				x	X	95	m	O+	Rectum	AC				

Patients	Section	Description of section	Types on section					Age	Gender	Blood group	Site	Cancer type	pT	pN	pM	Dukes
			N	HP	LGD	HGD	C									
J7	J7-A	Polypectomy	x	x				68	m	NK	Ascending colon					
J7	J7-B	Polypectomy	x	x	x			68	m	NK	Sigmoid colon					
J7	J7-C	Polypectomy	x		x			68	m	NK	Sigmoid colon					
J7	J7-D	Polypectomy	x		x	x		68	m	NK	Sigmoid colon					
J7	J7-E	Polypectomy						68	m	NK	Sigmoid colon					
J7	J7-F	Polypectomy			x	x		68	m	NK	Sigmoid colon					
J8	J8-A	Polypectomy			x	x		66	m	NK	Sigmoid colon					
J9	J9-A	Polypectomy	x					60	m	NK	Sigmoid colon					
J10	J10-A	Polypectomy		x				60	m	NK	Sigmoid colon					
J10	J10-A	Polypectomy		x				60	m	NK	Rectum					
J10	J10-B	Polypectomy		x				60	m	NK	Rectum					
J10	J10-C	Polypectomy		x				60	m	NK	Transverse colon					
J11	J11-A	Polypectomy	x	x				62	f	NK	Transverse colon					
J11	J11-B	Polypectomy	x	x				62	f	NK	Sigmoid colon					
J11	J11-C	Polypectomy	x	x	x			62	f	NK	Descending colon					

Patients	Section	Description of section	Types on section					Age	Gender	Blood group	Site	Cancer type	pT	pN	pM	Dukes
			N	HP	LGD	HGD	C									
J12	J12-A	Polypectomy		x				66	m	NK	Descending colon					
J12	J12-B	Polypectomy	x	x				66	m	NK	Descending colon					
J13	J13-A	Polypectomy			x			62	m	NK	Descending colon					
J14	J14-A	Biopsy of polyp				x		60	m	B+	Sigmoid colon					
J15	J15-A	Polypectomy			x			66	m	O+	Caecum					
J15	J15-B	Polypectomy			x			66	m	O+	Ascending colon					
J15	J15-C	Polypectomy				x		66	m	O+	Rectum					
J16	J16-A	Polypectomy			x			70	m	NK	Descending colon					
J16	J16-B	Polypectomy	x		x			70	m	NK	Sigmoid colon					
J16	J16-C	Polypectomy	x			x		70	m	NK	Rectum					
J17	J17-A	Polypectomy			x			64	m	NK	Sigmoid colon					
J17	J17-B	Polypectomy			x	x		64	m	NK	Sigmoid colon					
J18	J18-A	Polypectomy	x		x			75	m	O+	Caecum					
J18	J18-B	Polypectomy			x			75	m	O+	Rectum					
J19	J19-A	Polypectomy			x			75	f	A+	Sigmoid colon					
J20	J20-A	Normal mucosal biopsy	x					67	f	A+	Sigmoid colon					

Patients	Section	Description of section	Types on section					Age	Gender	Blood group	Site	Cancer type	pT	pN	pM	Dukes
			N	HP	LGD	HGD	C									
J20	J20-B	Normal mucosal biopsy	x					67	f	A+	Rectum					
J20	J20-C	Polypectomy			x			67	f	A+	Sigmoid colon					
J21	J21-A	Polypectomy	x	x	x			79	m	A+	Sigmoid colon					
J22	J22-A	Biopsy of cancer	x				x	64	m	NK	Rectum	AC				
J23	J23-A	Polypectomy	x		x	x		68	m	A+	Sigmoid colon					
J24	J24-A	Polypectomy	x		x			62	m	NK	Transverse colon					
J24	J24-B	Polypectomy	x					62	m	NK	Transverse colon					
J24	J24-C	Polypectomy	x		x			62	m	NK	Sigmoid colon					
J25	J25-A	Normal mucosal biopsy	x					65	m	NK	Transverse colon					
J25	J25-B	Polypectomy	x		x			65	m	NK	Transverse colon					
J25	J25-C	Polypectomy			x	x		65	m	NK	Sigmoid colon					
J25	J25-D	Polypectomy		x				65	m	NK	Sigmoid colon					
J26	J26-A	Polypectomy	x		x			68	f	NK	Ascending colon					
J26	J26-B	Polypectomy			x			68	f	NK	Sigmoid colon					

Patients	Section	Description of section	Types on section					Age	Gender	Blood group	Site	Cancer type	pT	pN	pM	Dukes
			N	HP	LGD	HGD	C									
J27	J27-A	Polypectomy	x		x	x		64	f	NK	Sigmoid colon					
J27	J27-B	Polypectomy	x	x				64	f	NK	Rectum					
J28	J28-A	Polypectomy	x		x			75	m	A+	Transverse colon					
J28	J28-B	Polypectomy	x		x	x		75	m	A+	Sigmoid colon					
J28	J28-C	Polypectomy		x				75	m	A+	Sigmoid colon					
J28	J28-D	Polypectomy			x			75	m	A+	Rectum					
J29	J29-A	Polypectomy			x			58	m	NK	Sigmoid colon					
J29	J29-B	Polypectomy	x		x	x		58	m	NK	Sigmoid colon					
J30	J30-A	Polypectomy		x				60	f	A+	Rectum					
J31	J31-A	Polypectomy	x	x				60	f	NK	Sigmoid colon					
J32	J32-A	Normal mucosal biopsy	x					62	m	A+	Ascending colon					
J32	J32-B	Polypectomy	x			x		62	m	A+	Descending colon					
J33	J33-A	Polypectomy: Cancer			x	x	x	66	f	A+	Descending colon	AC	pT1			
J34	J34-A	Resection specimen: distal resection margin	x					76	m	O+	Ascending colon					

Patients	Section	Description of section	Types on section					Age	Gender	Blood group	Site	Cancer type	pT	pN	pM	Dukes
			N	HP	LGD	HGD	C									
J34	J34-B	Resection specimen: proximal resection margin	x					76	m	O+	Ascending colon					
J34	J34-C	Resection specimen: larger polyp	x			x		76	m	O+	Ascending colon					
J34	J34-D	Resection specimen: tumour	x				x	76	m	O+	Ascending colon	AC	pT4b	pN2	pM0	C2
J35	J35-A	Resection specimen: tumour	x	x	x	x	x	71	f	O+	Transverse colon	AC	pT1	pN0	pM0	A
J36	J36-A	Resection specimen: proximal and distal resection margin	x					77	m	A+	Ascending colon					
J36	J36-B	Resection specimen: tumour					x	77	m	A+	Ascending colon	AC	pT4a	pN1	pM0	C1
J37	J37-A	Resection specimen: distal resection margin	x					78	m	A-	Transverse colon					

Patients	Section	Description of section	Types on section					Age	Gender	Blood group	Site	Cancer type	pT	pN	pM	Dukes
			N	HP	LGD	HGD	C									
J37	J37-B	Resection specimen: large bowel polyp levels	x		x			78	m	A-	Transverse colon					
J37	J37-C	Resection specimen: tumour	x				x	78	m	A-	Transverse colon	AC	pT4a	pN0	pM0	B
J38	J38-A	Resection specimen: tumour limit+lymph node	x					83	f	A+	Sigmoid colon					
J38	J38-B	Resection specimen: tumour &serosa					x	83	f	A+	Sigmoid colon	AC	pT4a	pN0	pM0	B
J39	J39-A	Resection specimen: distal resection margin	x					77	m	A-	Ascending colon					
J39	J39-B	Resection specimen: polyp	x	x				77	m	A-	Ascending colon					
J39	J39-C	Resection specimen: tumour					x	77	m	A-	Ascending colon	AC	pT4a	pN2	pM0	C2

Patients	Section	Description of section	Types on section					Age	Gender	Blood group	Site	Cancer type	pT	pN	pM	Dukes
			N	HP	LGD	HGD	C									
J40	J40-A	Resection specimen: proximal resection margin	x					77	m	A+	Rectum					
J40	J40-B	Resection specimen: proximal resection margin	x				x	77	m	A+	Rectum	AC	pT4a	pN1	pM0	C1
J40	J40-C	Resection specimen: LS of distal resection margin with tumour	x					77	m	A+	Rectum					
J40	J40-D	Resection specimen: Polyp at resection margin	x	x				77	m	A+	Rectum					
J40	J40-E	Resection specimen: polyp 10mm from resection margin	x	x				77	m	A+	Rectum					

Patients	Section	Description of section	Types on section					Age	Gender	Blood group	Site	Cancer type	pT	pN	pM	Dukes
			N	HP	LGD	HGD	C									
J40	J40-F	Resection specimen: tumour	x			x		77	m	A+	Rectum					
J40	J40-G	Resection specimen: tumour	x	x	x		x	77	m	A+	Rectum					
J40	J40-H	Resection specimen: tumour					x	77	m	A+	Rectum					
J41	J41-A	Resection specimen: tumour	x				x	77	m	O+	Ascending colon	AC	pT2	pN0	pM0	A
J41	J41-B	Resection specimen: tumour	x			x	x	77	m	O+	Ascending colon					
J41	J41-C	Resection specimen: polyp	x	x				77	m	O+	Ascending colon					
J41	J41-D	Resection specimen: colon end/donut	x					77	m	O+	Ascending colon					
J42	J42	Polypectomy	x	x				58	m	NK	Transverse colon					
J43	J43	Polypectomy	x		x			71	f	A-	Descending colon					
J44	J44	Polypectomy	x		x			55	m	NK	Sigmoid colon					

Patients	Section	Description of section	Types on section					Age	Gender	Blood group	Site	Cancer type	pT	pN	pM	Dukes
			N	HP	LGD	HGD	C									
J45	J45	Polypectomy	x		x			88	f	A+	Caecum					
J46	J46	Normal mucosal biopsy	x					72	f	O+	Transverse colon					
J47	J47	Polypectomy	x		x			64	m	NK	Rectum					

AC-adenocarcinoma; NK- not known.
